# Progressive hypoventilation due to mixed CD8^+^ and CD4^+^ lymphocytic polymyositis following tremelimumab - durvalumab treatment

**DOI:** 10.1186/s40425-017-0258-x

**Published:** 2017-07-18

**Authors:** Sooraj John, Scott J. Antonia, Trevor A. Rose, Robert P. Seifert, Barbara A. Centeno, Aaron S. Wagner, Ben C. Creelan

**Affiliations:** 1 Morsani College of Medicine, University of South Florida, 12901 Bruce B. Downs Blvd., Tampa, FL 33612 USA; 2 Department of Immunology, H. Lee Moffitt Cancer Center and Research Institute, 12902 Magnolia Dr., Tampa, FL 33612 USA; 3 Department of Diagnostic Radiology, H. Lee Moffitt Cancer Center and Research Institute, 12902 Magnolia Dr., Tampa, FL 33612 USA; 4 Department of Pathology and Cell Biology, University of South Florida, 12901 Bruce B. Downs Blvd., MDC 11, Tampa, FL 33612 USA; 5 Department of Pathology, H. Lee Moffitt Cancer Center and Research Institute, 12902 Magnolia Dr., Tampa, FL 33612 USA; 6 Orlando Health Pathology, 1414 Kuhl Ave., MP 44, Orlando, FL 32806 USA; 7 Department of Thoracic Oncology, H. Lee Moffitt Cancer Center and Research Institute, 12902 Magnolia Drive, Tampa, FL 33612 USA

**Keywords:** Immune-related adverse event, Non-small cell lung cancer, Programmed death protein 1, Programmed death-ligand 1, Cytotoxic T-lymphocyte-associated-protein 4, Immune checkpoint inhibitor, Myasthenia gravis, Striated muscle antibody, MEDI4736

## Abstract

**Background:**

The combination of CTLA-4 and PD-L1 inhibitors has a manageable adverse effect profile, although rare immune-related adverse events (irAE) can occur.

**Case presentation:**

We describe an autoimmune polymyositis following a partial response to combination tremelimumab and durvalumab for the treatment of recurrent lung adenocarcinoma. Radiography revealed significant reduction in all metastases; however, the patient developed progressive neuromuscular hypoventilation due to lymphocytic destruction of the diaphragmatic musculature. Serologic testing revealed a low level of de novo circulating antibodies against striated muscle fiber. Immunohistochemistry revealed type II muscle fiber atrophy with a mixed CD8^+^ and CD4^+^ lymphocyte infiltrate, indicative of inflammatory myopathy.

**Conclusions:**

This case supports the hypothesis that muscle tissue is a target for lymphocytic infiltration in immune checkpoint inhibitor-associated polymyositis. Further insights into the autoimmune mechanism of PM will hopefully contribute to the prevention and treatment of this phenomenon.

**Electronic supplementary material:**

The online version of this article (doi:10.1186/s40425-017-0258-x) contains supplementary material, which is available to authorized users.

## Background

Combination therapy with anti-cytotoxic T-lymphocyte-associated-protein 4 (CTLA-4) and anti-programmed death ligand 1 (PD-L1) monoclonal antibodies holds incredible potential for the treatment of solid tumors [1]. Despite their robust activity, these immune checkpoint inhibitors can have rare but important immune-related adverse events (irAEs). Specifically, CTLA-4 inhibitors have been associated with irAEs in most organ systems, including enterocolitis, hepatitis, and endocrinopathy [2]. Among these irAEs, there are several cases of inflammatory myopathies [3].

Polymyositis, dermatomyositis, and inclusion body myositis (IBM) are among a group of inflammatory myopathies associated with muscle weakness and inflammatory infiltrates within skeletal muscle. Polymyositis (PM) is a subacute myopathy which differs from other subgroups by perifascicular atrophy and the absence of vacuoles [4]. In animal models, PM is induced by clonal expansion of cluster of differentiation (CD) 8^+^ cells of specific T cell receptor (TCR) families targeting muscle tissue. High levels of interferon gamma (IFN-γ) lead to up-regulation of MHC class I in myotubes, even in areas far from sites of inflammation [5]. Perforin-dependent cytotoxicity mediated by CD8^+^ T cells causes muscle fiber atrophy and necrosis [6]. Diaphragmatic weakness is one of the most prominent and life-threatening consequences of autoimmune myositis [7].

Herein we describe a patient treated with a single infusion of combination anti-CTLA-4 and anti-PD-L1 antibody, and subsequently incurred a fatal destructive lymphocytic myositis involving the inspiratory muscles. We performed a retrospective analysis of this subject with the primary objective to further characterize the immunopathology of this rare adverse event.

## Case presentation

**Fig. 1:**
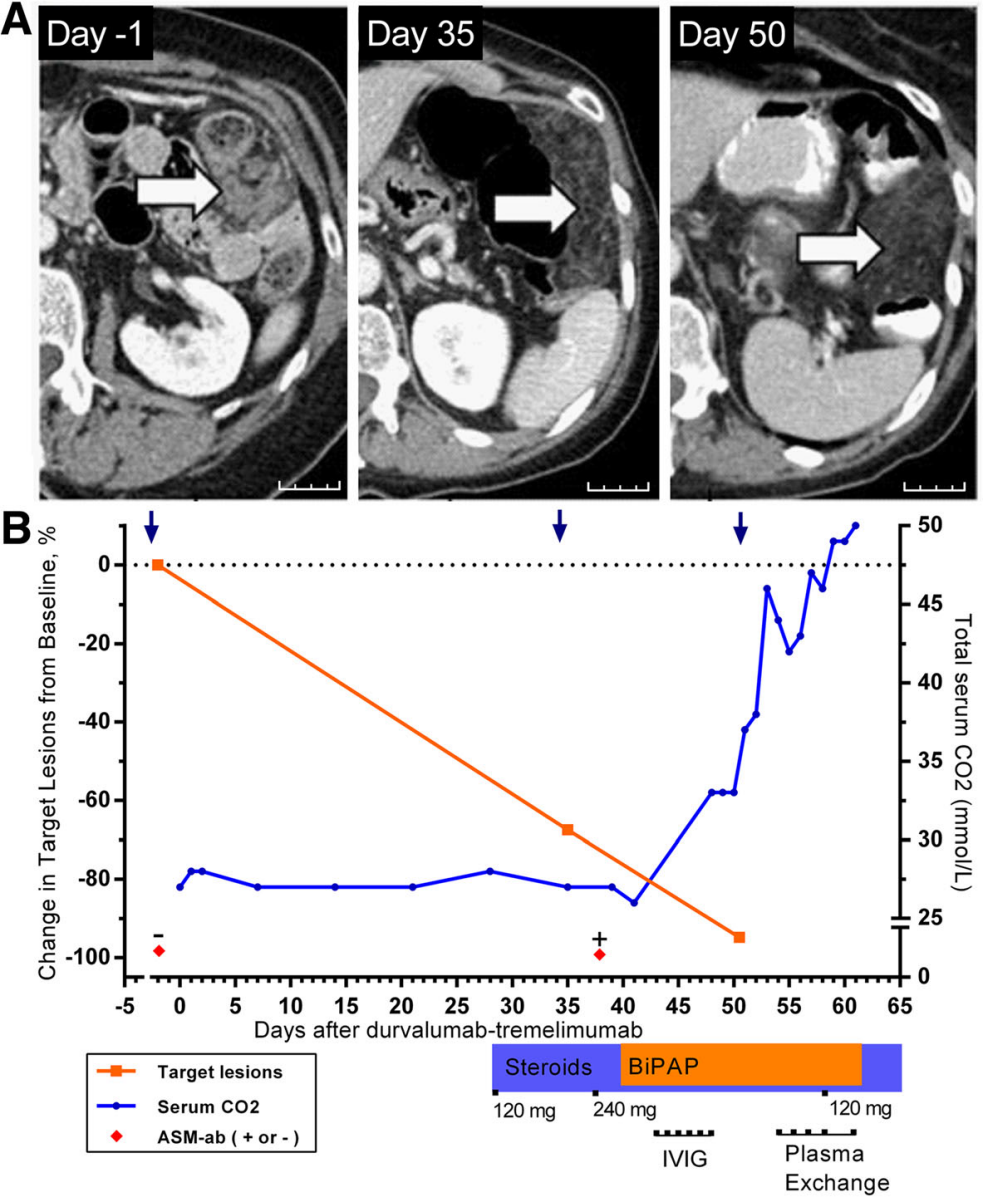
**a** Change in representative omental metastasis on CT scan after treatment with durvalumab- tremelimumab on Day 1. *Arrows* indicate omental metastasis replaced by fatty tissue. *Scale bar* indicates 25 mm. **b** Clinical course of patient until death from neuromuscular respiratory failure on Day 65. Serum CO_2_ retention is indicative of hypoventilation

A 64-year-old female former smoker presented with lung adenocarcinoma originally treated with right pneumonectomy. Follow-up imaging four years later revealed a recurrence involving the omentum, confirmed by biopsy. Primer-extension mass spectrometry genotyping of the tumor revealed a KRAS ^G12C^ mutation, and immunohistochemistry showed no PD-L1 expression in tumor cells. She was seronegative for HIV and viral hepatitis. The patient expressed a preference for immunotherapy instead of palliative chemotherapy. She began a phase 1 clinical trial (NCT02000947) consisting of tremelimumab (1 mg/kg) and durvalumab (10 mg/kg) IV over 1 h on Day 1 [8]. At Day 28, she reported progressive dysphagia and a barium esophagram demonstrated hypomotility. Soon thereafter, she developed respiratory acidosis. Magnetic resonance imaging (MRI) was normal, and lumbar puncture revealed normal cerebrospinal fluid (CSF) cell count with negative cytology. Computed tomography (CT) scan demonstrated a significant reduction in the size of the omental metastases (Fig. 1). Further tremelimumab -durvalumab was stopped, and she was treated empirically with high-dose prednisone at 120 mg/day. Despite this, she experienced progressive neuromuscular hypoventilation requiring continuous bilevel positive airway pressure (BiPAP) ventilation, with declined forced vital capacity (0.73 L) and impaired negative inspiratory force (−41 cm H_2_O). Intravenous polyvalent immunoglobulin (1 g/kg, Day 42–46), plasma exchange (Days 54–61), and pyridostigmine (Day 49–65) were added, with no effect. Eventually she requested de-escalation to comfort care, and died peacefully after withdrawal of BiPAP support on Day 65.

Although the patient was initially suspected to have a drug-related myasthenia syndrome, consequent testing for acetylcholine receptor (AChR) immunoglobulin (IgG), voltage gated calcium channel (VGCC) IgG, and muscle-specific kinase protein (MuSK) IgG was negative. Anti-striated muscle (ASM) IgG was detected at a low titer of 1:40; it was not detected in archived pretreatment serum. Since the ASM IgG was only detectable at low titer, we could not conclude a diagnosis based solely upon the serology findings.

**Fig. 2:**
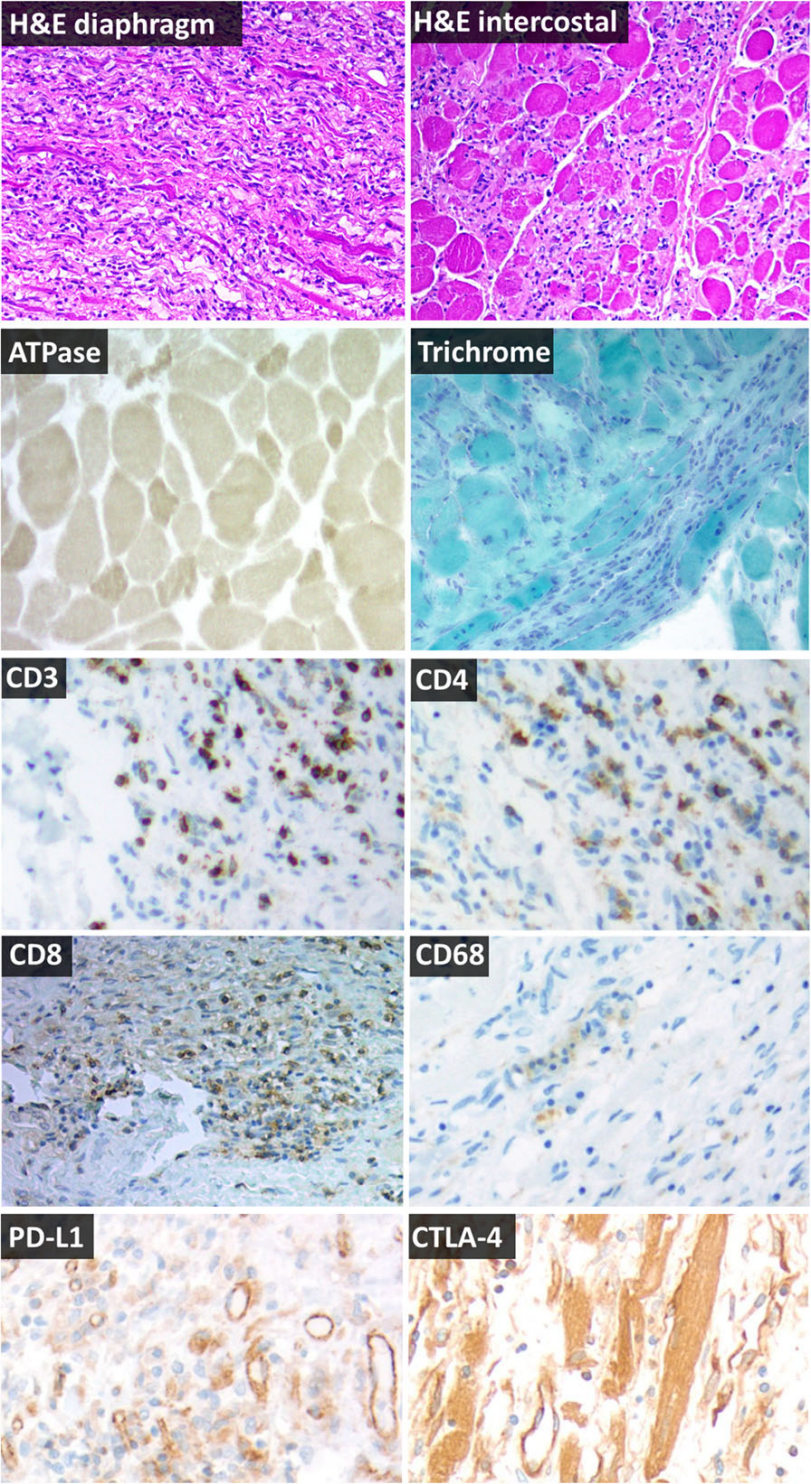
Representative immunohistochemistry of inspiratory muscles at autopsy, 66 days after tremelimumab-durvalumab treatment. Hematoxylin and eosin (H&E) sections show inflammatory myopathy in the diaphragm and intercostal muscles without rimmed vacuoles and without perifascicular atrophy, consistent with polymyositis. A mononuclear infiltrate is present which invades otherwise normal myofibers and completely effaces the background muscle fiber architecture in some areas. ATPase shows intense staining in small fibers compared to surrounding lighter, normal sized fibers in preserved areas of muscle, indicative of type II fiber atrophy. Trichrome shows mildly increased connective tissue, but shows no rimmed vacuoles, rods, or other inclusions. T cell co-receptor staining (CD3, CD4, CD8) revealed a mixed T-cell infiltrate which often completely effaced the myofascicular architecture. CD68 highlights necrotic myofibers scattered within the larger inflammatory infiltrate. PD-L1 expression was observed in blood vessels of dying muscle. Weak CTLA-4 expression was detected in necrotic myofibers. All images are 100× magnification

Upon autopsy, gross examination showed mild atrophy of the intercostal and diaphragm muscle. There was no evidence of interstitial lung disease. An additional file describes the methods used (Additional file 1) and controls used (Additional file 2). Microscopic examination of the inspiratory musculature showed an inflammatory mononuclear infiltrate most pronounced in the diaphragm, with only scattered preserved fibers (Fig. 2). Immunohistochemistry showed no increased lipid stores, and an attenuated mosaic pattern with type II fiber specific atrophy. Transactive response (TAR) DNA-binding protein 43 and trichrome evaluation were negative for inclusion bodies [9]. Mononuclear cells infiltrating the diaphragm and intercostal muscle consisted of a mixed phenotype of CD8^+^ and CD4^+^ lymphocytes. CD68^+^ macrophages were also observed in necrotic myofibers [10]. No residual metastases were identified on gross examination, indicating a complete pathological response to tremelimumab-durvalumab.

## Discussion

As CTLA-4 and PD-1 axis inhibition becomes adopted for more cancer types, rare irAE presentations are becoming more common. Severe PM has been reported with monoclonal antibodies against both CTLA-4 and PD-1 (Table 1). This disease classically affects the proximal muscles and in severe cases, such as this one, is associated with dysarthria and dysphagia [11]. Although diaphragmatic PM has been previously reported [9], our case is prominent for its acute onset following immune-checkpoint inhibitor therapy.

**Table 1:** Select cases of severe myositis associated with CTLA-4 and/or PD-1 axis inhibitors

Drug(s)	Description	↑CK?	ASM IgG?	Onset (wks)	Treatment	Trial	Reference
Ipilimumab + nivolumab	Polymyositis with respiratory involvement	Yes	Yes	3	Corticosteroid, infliximab, IVIG, Pex	NCT01928394	[35]
Nivolumab	Myositis with respiratory failure	Yes	–	7	Corticosteroid	–	[36]
Nivolumab	Myasthenia crisis and polymyositis requiring ventilation	Yes	No	2	Corticosteroid, Pex, IVIG pyridostigmine	–	[19]
Pembrolizumab	Polyarticular tenosynovitis and proximal myositis	No	–	56	Sulfasalazine	NCT01295827	[37]
Pembrolizumab	Exacerbation of preexisting myositis	Yes	–	1	IVIG	–	[38]
Ipilimumab	Dermatomyositis	Yes	–	2	Corticosteroid	–	[39]
Pembrolizumab / ipilimumab	Rhabdomyolysis associated with hypothyroidism	Yes	–	6	Levothyroxine	–	[40]
Ipilimumab	Combined myasthenia and myositis with AChR	Yes	Yes	7	Corticosteroid, IVIG	–	[26]
Ipilimumab	Retrobulbar weakness with proximal myositis.	Yes	Yes	7	Corticosteroid, IVIG	–	[3]
Ipilimumab	Orbital myositis associated with ipilimumab	–	–	12	Corticosteroid	–	[41]
Tremelimumab + durvalumab	Described in text.	No	Yes	4	Corticosteroid, Pex, IVIG	NCT02000947	

*Abbreviations: ASM*, anti-striated muscle antibody, *NCT* national clinical trials identifier number, *Pex* plasma exchange, *IVIG* intravenous immunoglobulin G, *AChR* acetylcholine receptor antibody, *CK* creatine kinase, *wks* weeks

The pathogenesis of idiopathic PM has intrinsic mechanistic overlap with the action of CTLA-4 blockade. Anti-CTLA-4 monoclonal antibody treatment increases IFN-γ production in draining lymph nodes [12], which may induce higher MHC class I expression on adjacent cells. Like the drug-related PM reported here, idiopathic PM is identified by disorganized muscle fibers of variable sizes coupled with endomysial T cell infiltrates [13]. CD8^+^ cells preferentially invade fibers which express MHC class I and trigger necrosis via the perforin pathway [4]. Although terminally differentiated, these CD8^+^ T cell infiltrates are predominantly CD28^null^ [14]. In patients, this CD28^null^ phenotype correlates with resistance to corticosteroid therapy [15]. Thus, aberrant B7-family receptor function appears to have a contributory role in idiopathic PM, not unlike the CTLA-4 drug-related PM described.

Targeting of muscle fiber by autoreactive T cells has been proposed as the pathogenesis of idiopathic PM [16]. Muscle-related antigens have been recognized in TCRs sequenced from idiopathic PM lesions [17], and a focused T cell repertoire is observed in these lesions [18]. Consistent with idiopathic PM, TCR sequencing of inflamed muscle in a nivolumab-related PM case revealed a clonally expanded T cell population in the muscle, as compared to either blood or tumor [19]. Like most irAEs associated with CTLA-4 or PD-1 inhibition, PM is also a recognized complication of up to 7.8% of allogeneic stem cell transplants, due to graft-versus-host disease (GvHD) [20]. It occurs after onset of hematopoietic full chimerism, and is associated with an increase in circulating CD8^+^ T cells. Muscle biopsy often reveals a mononuclear infiltrate of donor T cells and macrophages at endomysial sites, as in our case [21]. However, GvHD PM usually responds promptly to high-dose corticosteroids or cyclosporine [22].

The PD-1 axis is also implicated in the development of idiopathic myositis. Skeletal muscle cells normally express PD-L1, which induces T cell anergy. PD-L1 had a immunoprotective role against myositis in coculture experiments of MHC class I/II labeled myoblasts with CD4^+^ or CD8^+^ T cells [23]. Thus, PD-1 or PD-L1 monoclonal antibody may theoretically contribute to the autoimmune myositis cascade by attenuating the protection of skeletal muscle cells against autoreactive T cells [24].

The pathological findings in our case seem most indicative of PM, although a seronegative myasthenia overlap condition may also have been present. A Murine B6 model suggests that anti-CTLA-4 treatment stimulates AChR auto-antibody production and enhances the T cell response to provoke severe autoimmune myasthenia gravis [25]. Several cases of myasthenia gravis have been reported with ipilimumab [26, 27], as well as ipilimumab-nivolumab [28].

Optimal treatment of KRAS-mutant lung adenocarcinoma remains an emerging field. At present, therapy with PD-L1 axis blockade appears to be at least as effective in KRAS-mutant lung adenocarcinomas, compared to wild-type [29, 30]. Although KRAS point mutations do not appear to result in highly antigenic proteins, there has been preclinical success in inducing T-cell responses against Ras-associated epitopes [31]. In addition, TCRs reactive to the mutant KRAS^G12D^ peptide have been isolated within CD8+ TIL cultured from colon adenocarcinoma [32, 33]. Likewise, TCRs reactive to KRAS^G12D/G12V^ peptides have been isolated from immunized, HLA-specific transgenic mice [34]. Thus, adoptive cell transfer may be a viable treatment option for the proportion of KRAS-mutant patients who do not respond to PD-L1 / CTLA-4 blockade.

This study was conducted post-mortem, and is characterized by important limitations. Unfortunately, electromyography was not conducted. In addition, sufficient volumes of blood to determine the specific epitope of striated muscle protein were not collected, or determine the putative autoantigens involved. Absence of properly preserved PBMCs precluded lymphocyte reactivity testing. In addition, no blood samples suitable for creatine kinase measurement were collected. The ASM IgG was only weakly positive in our case, which is inconclusive as a standalone finding. Nonetheless, PM was unmistakable on pathologic examination.

## Conclusion

PM remains a rare but important irAE associated with anti-CTLA-4 and PD-L1 combination therapy. The case reviewed in this report supports the hypothesis that muscle tissue is a target for lymphocytic infiltration in tremelimumab - durvalumab associated PM. Fatality was an unfortunate outcome in this case, perhaps tied in part to delayed recognition. In similar cases, prompt initiation of corticosteroids and additional measures have limited the severity of PM. Further insights into the autoimmune mechanism of PM will hopefully contribute to the prevention and treatment of this phenomenon.

## Additional files

Additional file 1: Methods Section. This describes the methods used for deriving the clinical and pathological data presented. (DOCX 19 kb)

Additional file 2: Figure S1. This figure shows the positive and negative controls for immune cell immunohistochemistry. Benign lymph node tissue serving as positive (+) control for presence of T cell lineage cells (CD3, CD4, CD8) and macrophage lineage cells (CD68), as well as benign smooth muscle tissue serving as a negative (−) control for these antibodies. All images are 20× magnification. (TIFF 4756 kb)

## Abbreviations

AChR: Acetylcholine receptor
ASM: Anti-striated muscle
BiPAP: Bilevel positive airway pressure
CD: Cluster of differentiation
CO2: Carbon dioxide level
CSF: Cerebrospinal fluid
CT: Computed tomography
CTCAE: Common toxicity criteria for adverse events
CTLA-4: Cytotoxic T-lymphocyte-associated protein 4
GvHD: Graft-versus-host disease
H&E: Hematoxylin and eosin
HIER: High pH heat induced epitope retrieval
ICH –GCP: International conference on harmonization good clinical practice
IFN-γ: Interferon gamma
IHC: Immunohistochemistry
irAE: Immune-related adverse events
IV: Intravenous
IVIG: Intravenous immunoglobulin G
KRAS: Kirsten rat sarcoma viral oncogene homolog
MHC: Major histocompatibility
MRI: Magnetic resonance imaging
MuSK: Muscle specific kinase protein
NCT: National clinical trials
PBMCs: Peripheral blood mononuclear cells
PD-1: Programmed cell death protein 1
PD-L1: Programmed death ligand 1
Pex: Plasma exchange
PM: Polymyositis
RECIST: Response evaluation criteria in solid tumor
TAR DP-43: Transactive response DNA binding protein 43 kDa
TCR: T cell receptor
VGCC: Voltage gated calcium channel
wks: Weeks
