# Supplementary material for: Progressive hypoventilation due to mixed CD8+ and CD4+ lymphocytic polymyositis following tremelimumab - durvalumab treatment
Source: J Immunother Cancer. 2017 Jul 18;5:54. doi: 10.1186/s40425-017-0258-x (PMC5514517; doi:10.1186/s40425-017-0258-x)
Supplement: Supplementary file 1 — Methods Section. This describes the methods used for deriving the clinical and pathological data presented. (DOCX 19 kb) [file 40425_2017_258_MOESM1_ESM.docx]

**Additional file 1**

***Clinical study:***

This patient was treated on an open-label, single-center, phase I study using a modified 3+3 dose-escalation design, which has been previously reported [8]. Tremelimumab and durvalumab was administered starting on day 1, once every cycle, with a cycle every 28 days. This study was conducted at the H Lee Moffitt Cancer Center (Tampa, FL), and the case report was approved by local institutional review board. It was performed in accordance with the Declaration of Helsinki and the International Conference on Harmonization Good Clinical Practice (ICH –GCP) guidelines. Informed consent from the patient was previously obtained for research, including use of tissue for research. The primary objective was to characterize the pathogenesis of the adverse event.

***Serology:***

The archived pretreatment serum sample had been processed for the clinical trial in a serum separator (SST) tube of 0.5 mL aliquot, centrifuged, and frozen at −20°C. These samples were intended for batch testing related to the clinical trial. For the striational muscle antibody assay, specimen stability in frozen storage has a preferred optimal duration of 360 days or less, provided there are no repeated freeze/thaw cycles. In our case, the archival pretreatment serum had been frozen in storage for approximately 47 days. The Day +37 sample was also collected in an SST, centrifuged, and refrigerated at 2- 8 °C prior to testing. Testing was performed on patient serum diluted in PBS.

Reagents included anti-striated muscle antibody (ASM-ab) (ARUP: 0050746), acetylcholine receptor (AChR) antibody (ARUP: 0099580), AChR bind antibody (ARUP: 0080009), anti-voltage gated calcium channel (VGCC) antibody (KRONUS: KR6560), and anti-muscle specific kinase (MuSK) antibody (Athena: 482). Test slides were incubated in a moist chamber at room temperature and then washed x2 in phosphate buffered saline (PBS) chamber. One drop of conjugate was applied to each antigen well and then samples were incubated again in a moist chamber at room temperature and washed x2 in PBS chamber. Samples were then mounted and read with a fluorescence microscope. Results were expressed antibody titer, specifically the greatest dilution at which the optical density of the reaction product is >1.50 x the mean value of 4 normal control sera.

***Computed Tomography (CT)***

Axial Images were obtained on a standard-definition multi-slice 64-detector row scanner (Siemens Medical Solutions, Erlangen, Germany). Slices of 2.5 mm thickness through the chest, abdomen and pelvis were collected after the administration of non-ionic iohexol intravenous contrast (Omnipaque; GE healthcare).

Tumor measurement adhered to RECIST v1.1 criteria, with a procedural modification. Since three separate omental tumor nodules were measurable, all were included as target lesions.

***Immunohistochemistry:***

Autopsy tissue was collected and preserved in formalin. Suitable areas were selected by a pathologist and sectioned into 5 µm slides for immunohistochemistry. CD3 (Cell Marque, rabbit polyclonal), CD4 (Genetex, mouse monoclonal), CD8 (Cell Marque, mouse monoclonal), CD68 (Ventana, mouse monoclonal), PD-L1 (Sino, rabbit polyclonal), CTLA-4 (Santa Cruz, mouse monoclonal) were performed with Dako Link 48 autostainer after high pH heat induced epitope retrieval (HIER) digestion. Formalin fixed paraffin embedded tissue sections run through Leica Jung Autostainer XL using Program 1 (routine H&E stain) with Eosin Y (Sigma-Aldrich: E4382) and Harris Hematoxylin (Poly Scientific: S212) reagents.

For CD3, CD4, CD8, and CD68 glycoprotein antibodies, positive controls were performed upon benign lymph node tissue obtained using the same fixation and retrievals methods as described above. Likewise, negative controls were performed upon benign smooth muscle tissue obtained using identical methodology. These controls are reported in **Supplemental Figure 1**.
*For Gomori’s trichrome:* fresh, unfixed snap frozen striated muscle specimens were incubated in Harris Hematoxylin and then rinsed with water. Samples were stained in Gomori’s trichrome (Fast Green, FCF - Mallinckrodt: E605; Chromotrope 2R - Sigma-Aldrich: C-3143; Phosphotungstic acid - Mallinckrodt: 2824-01; Glacial Acetic Acid - Macron: 8817; de-ionized water (DIW)) adjusted to pH 3.4 using 1N NaOH.

*For ATPase:* fresh, unfixed striated muscle specimens snap frozen in 2-Methylbutane, sectioned adjusted to pH 9.4 with 0.1N HCL, incubated (2% Sodium Barbital; 2% Calcium Chloride; DIW; Adenosine 5’ Triphosphate - MP Biomedicals LLC: 05213412) for 20 min. and rinsed x 3, then briefly bathed in 1% yellow ammonium sulfate (Sigma Aldrich: 12135-76-1).
