# Supplementary figures and images for: Progressive hypoventilation due to mixed CD8+ and CD4+ lymphocytic polymyositis following tremelimumab - durvalumab treatment
Source: J Immunother Cancer. 2017 Jul 18;5:54. doi: 10.1186/s40425-017-0258-x (PMC5514517; doi:10.1186/s40425-017-0258-x)

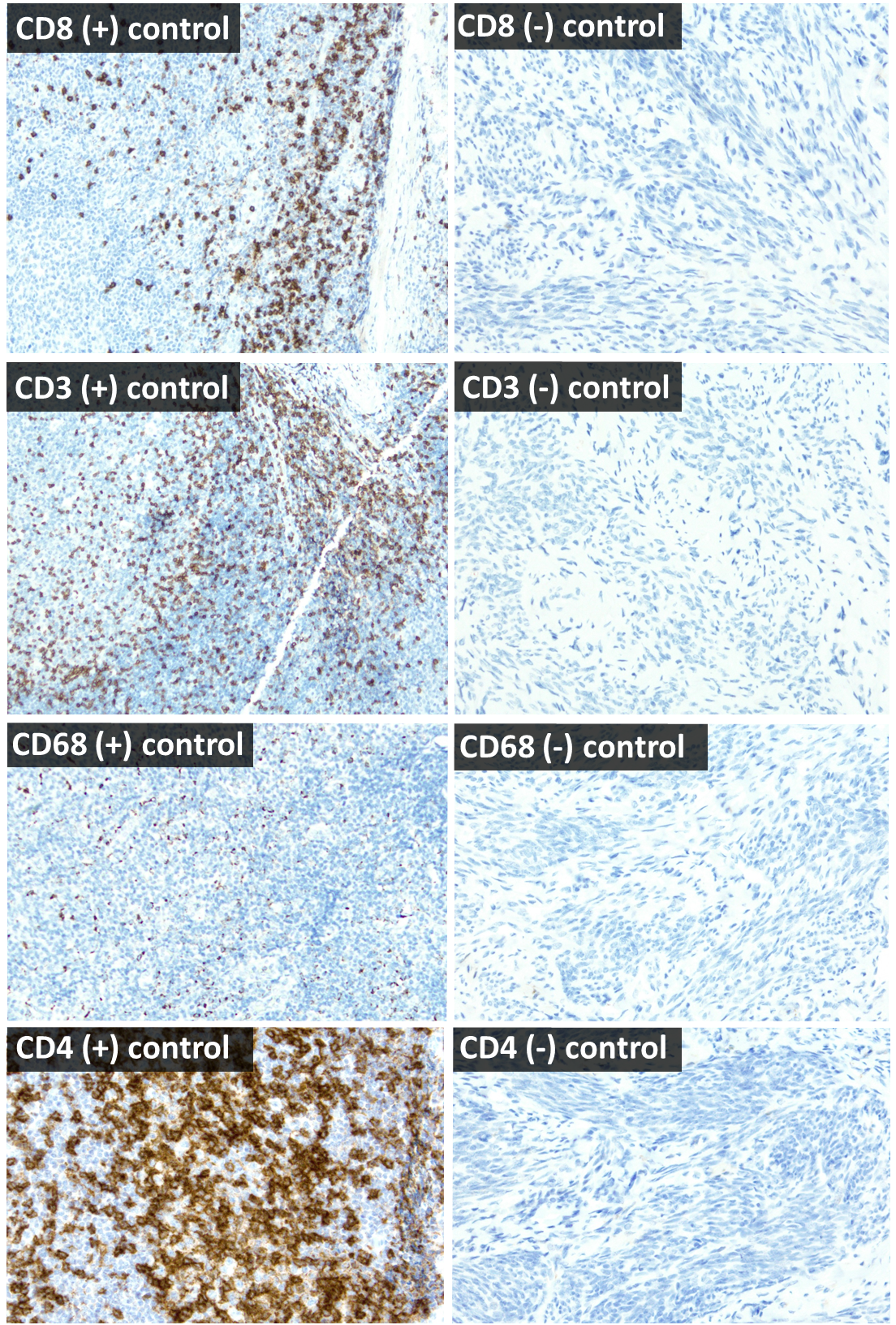

Supplement: Supplementary file 2 — This figure shows the positive and negative controls for immune cell immunohistochemistry. Benign lymph node tissue serving as positive (+) control for presence of T cell lineage cells (CD3, CD4, CD8) and macrophage lineage cells (CD68), as well as benign smooth muscle tissue serving as a negative (−) control for these antibodies. All images are 20× magnification. (TIFF 4756 kb) [file 40425_2017_258_MOESM2_ESM.tif]
